# Recent trends in exposure to secondhand smoke in the United States population

**DOI:** 10.1186/1471-2458-10-359

**Published:** 2010-06-23

**Authors:** Chieh-I Chen, Tanya Burton, Christine L Baker, Vera Mastey, David Mannino

**Affiliations:** 1 Pfizer Inc., 235 East 42nd Street, New York, NY 10017, USA; 2 Abt-Biopharma Solutions Inc. 181 Spring Street Lexington, MA 02421, 781.372.6652, USA; 3 University of Kentucky School of Medicine, 740 S. Limestone, K 528 Lexington, KY 40536, USA

## Abstract

**Background:**

Previous research using the National Health and Nutrition Examination Surveys (NHANES) data documented a significant downward trend in secondhand smoke (SHS) exposure between 1988 and 2002. The objective of this study was to assess whether the downward trend in exposure continued from 2001 through 2006.

**Methods:**

We analyzed data from the 2001-2006 NHANES to estimate exposure of nonsmokers to SHS. Geometric means of serum cotinine levels for all nonsmokers were computed.

**Results:**

Overall serum cotinine levels (95% Confidence Intervals) in 2001-2002, 2003-2004, and 2005-2006 were 0.06 ng/mL (0.05-0.07), 0.07 ng/mL (0.06-0.09), and 0.05 ng/mL (0.05-0.06), respectively. Subgroup analysis by age, gender, and race/ethnicity groups showed similar trends in cotinine levels. Children, males, and non-Hispanic Blacks had higher cotinine levels than adults, females, and non-Hispanic Whites and Mexican Americans, respectively. Insignificant *P *values from the Wald test indicate that serum cotinine levels did not differ over time.

**Conclusions:**

The long-term trend of declining exposure to SHS among nonsmokers appears to have leveled off. However, disparities noted in previous research persist today, with the young, non-Hispanic Blacks, and males experiencing higher levels of exposure.

## Background

Secondhand smoke (SHS) is a significant health hazard among nonsmokers in the United States. The National Toxicology Program has estimated that at least 250 chemicals in SHS are known to be toxic or carcinogenic. When nonsmokers are exposed to SHS, they are inhaling the same cancer-causing agents that current smokers inhale. Scientific evidence has documented SHS as a cause of major disease and death in the United States [1]. Secondhand smoke has been linked to 35,000 deaths from heart disease and 3000 deaths from lung cancer [2].

The health effects of SHS are particularly concerning in children. A large body of literature has linked SHS exposure to various respiratory illnesses and developmental problems in this group. Strong evidence has shown that children exposed to SHS are at higher risk for middle ear disease, asthma, respiratory symptoms, lung growth, and pulmonary function [1]. On an annual basis, more than 200,000 episodes of childhood asthma have been directly attributable to parental smoking [3]. In children, SHS exposure has also been associated with adverse behavioral outcomes, cognitive impairment, and poor school performance [1,4].

Secondhand smoke is defined as a mixture of smoke released by the burning end of a cigarette ("sidestream smoke") combined with cigarette smoke exhaled by the smoker ("mainstream smoke") [1]. Secondhand smoke is also referred to as environmental tobacco smoke. Since 1986, when the Surgeon General published a report establishing a causal link between SHS and major diseases, the body of supportive scientific literature has grown. In response to the growing body of scientific literature linking SHS with serious diseases, local and state governments across the nation have introduced a significant number of public health initiatives to reduce tobacco exposure, such as restricting smoking in work places and public settings (i.e., restaurants, bars, casinos, vehicles). These smoke-free legislative policies have proven to be effective for raising the public's awareness about the dangers of smoking and protecting the health of nonsmokers [1]. As an example, New York implemented a state law in 2003, requiring all indoor workplaces and public places to be smoke-free. One year following its implementation, self-reported SHS exposure at work decreased by 98% among nonsmoking employees of restaurants, bars, and bowling facilities, and their saliva cotinine levels decreased by 78% [5].

On a national level, SHS exposure among US nonsmokers has declined substantially since the late 1980 s [1,6]. Using the National Health and Nutrition Examination Surveys (NHANES), Pirkle et al [6] described SHS exposure over a period of 14 years between 1988 and 2002. The authors documented a substantial decline of approximately 70% in serum cotinine concentrations of nonsmokers during this period, although nonsmoking children, males, and non-Hispanic Blacks were found to have significantly higher serum cotinine concentrations than adults, females and non-Hispanic Whites and Mexican Americans, respectively.

Public health initiatives have had an important impact on the progress of tobacco control. Additionally, the decrease in the prevalence of cigarette smoking, from 28% in 1988 to 20.8% in 2006, has helped to accelerate the reduction of tobacco exposure [7].

The current manuscript builds on the earlier 1988-2002 findings from Pirkle et al. [6]. The study objectives were 2-fold: (1) to assess whether the downward trend in SHS exposure level has continued since 2002 and (2) to replicate, update, and expand upon the work of Pirkle et al. work of examining the cotinine levels of nonsmokers by using the most recently available NHANES data from 2001-2006.

## Methods

### Data source

Data were obtained from the NHANES conducted from 2001 to 2006. The NHANES was conducted by the National Center for Health Statistics of the Centers for Disease Control and Prevention (Atlanta, Georgia) and approved by the Institutional Review Board of the National Center for Health Statistics. The NHANES used a stratified, multistage, clustered probability design to select a nationally representative sample of the civilian, non-institutionalized US population. This study analyzed data over 3 time periods: 2001-2002, 2003-2004, and 2005-2006. Further details regarding the NHANES may be found elsewhere [8].

### Population selection

For comparison of NHANES data across time, we adopted methodology from previous publications by Pirkle et al to define non-smokers as individuals with a serum cotinine concentration ≤10 ng/mL. The study population included individuals aged 4 years and older at the time of their participation in the NHANES and who reported being non-Hispanic White, non-Hispanic Black, or Mexican American. A total of 19,890 participants were included in this study. The limit of detection in cotinine analysis was defined as individuals with a serum cotinine concentration at 0.05 ng/mL.

All survey participants (or their parent or guardian) provided written informed consent, answered health and nutrition questionnaires at home, and completed a comprehensive set of physical examinations in a mobile examination center. The serum cotinine analysis was performed on a blood sample drawn during the physical examination.

### Statistical analysis

SUDAAN statistical software (Research Triangle Institute, Research Triangle Park, North Carolina) was used to generate the variance estimates to account for the complex survey design. Data were analyzed using sampling weights to generate population estimates and adjust for the unequal probabilities of selection. The proportion of sample with a limit of detection of 0.050 ng/mL across the study time periods was generated. Given serum cotinine values have been shown to follow a log-normal distribution, [6] regression models for each survey year were performed using the log of serum cotinine as the dependent variable. The independent variables analyzed included: age (4-11, 12-19, 20+ years), gender, and race/ethnicity (non-Hispanic White, non-Hispanic Black, or Mexican American). Adults are defined as individuals 20 years old and above. Geometric means and 95% confidence intervals (CI) of serum cotinine were generated for all main level and 2-way interaction effects. The Wald test was used to test for a change over time overall and within each subgroup.

## Results

### Demographic characteristics

This analysis was limited to nonsmokers, defined as those with a serum cotinine concentration ≤10 ng/mL. Of the 31,509 participants in the 2001-2006 NHANES surveys, 27,143 had a serum cotinine concentration ≤10 ng/mL. Further exclusions based on age (below age of 4 years) and race/ethnicity ("Other") limited the final study sample to 19,890 participants (6942 in 2001-2002, 6377 in 2003-2004, and 6571 in 2005-2006). On average, 187 million children and adults in the United States were represented within each study period. The sample characteristics did not vary by study time period. After adjusting for the complex survey design in each of the 3 study time periods, on average, 72% of the nonsmokers were adults, 46% were male, and 76% were non-Hispanic White.

### Serum cotinine levels

**Figure 1:**
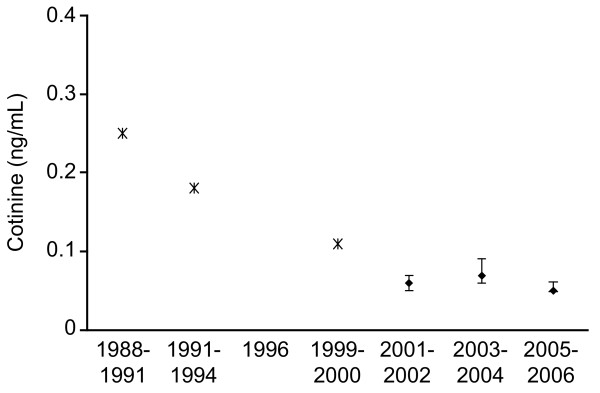
**Geometric means and 95% confidence intervals for nonsmokers in the United States from 1988 through 2006**. *Exposure data for 1998 through 2000 are derived from Pirkle et al [6] and embedded for comparison to current study findings.

The geometric means of serum cotinine levels of United States nonsmokers, overall and by subgroups (age, gender, and race/ethnicity) are presented in Table 1. The 3 time periods included in this table and subsequent figures are 2001-2002, 2003-2004, and 2005-2006. The geometric means of serum cotinine in overall nonsmokers were 0.06 ng/ml, 0.07 ng/ml, and 0.05 ng/ml, during 2001-2002, 2003-2004, and 2005-2006, respectively. Although the cotinine levels in 2005-2006 were either the same or lower than in 2001-2002, the insignificant *P *values, overall and within each subgroup, suggest that serum cotinine levels did not differ over time. Figure 1 shows the overall cotinine levels in longitudinal context from 1988 through 2006 by embedding previous data with our current findings [6]. The pattern of results within the age, gender, and race/ethnicity groups remained similar across the 3 time periods. Children, males, and non-Hispanic Blacks had higher cotinine levels than adults, females, and non-Hispanic White and Mexican Americans, respectively. The proportion of individuals with undetectable cotinine levels were 40%, 41%, and 45%, during 2001-2002, 2003-2004, and 2005-2006, respectively.

**Table 1:** Serum cotinine geometric means (95% confidence interval) for nonsmokers in United States populations 2001-2006

Geometric means of serum cotinine (ng/mL)	NHANES 2001-2002	NHANES 2003-2004	NHANES 2005-2006	*P *value
Overall	0.06 (0.05, 0.07)	0.07 (0.06, 0.09)	0.05 (0.05, 0.06)	0.12
Overall - Undetected	0.018 (0.016, 0.020)	0.018 (0.017, 0.020)	0.019 (0.018, 0.020)	0.75
Age: (years)				
4-11	0.11 (0.07, 0.15)	0.14 (0.07, 0.20)	0.08 (0.06, 0.10)	0.05
4-11 - Undetected	0.018 (0.015, 0.020)	0.018 (0.017, 0.020)	0.018 (0.018, 0.020)	0.84
12-19	0.09 (0.05, 0.12)	0.11 (0.08, 0.14)	0.08 (0.06, 0.10)	0.08
12-19 - Undetected	0.018 (0.016, 0.020)	0.018 (0.017, 0.020)	0.19 (0.018, 0.021)	0.59
20+	0.05 (0.04, 0.06)	0.06 (0.04, 0.07)	0.05 (0.04, 0.05)	0.29
20+ - Undetected	0.018 (0.016, 0.020)	0.018 (0.017, 0.020)	0.19 (0.018, 0.020)	0.79
Gender				
Male	0.07 (0.05, 0.09)	0.09 (0.07, 0.10)	0.07 (0.06, 0.07)	0.08
Male - Undetected	0.019 (0.016, 0.021)	0.020 (0.019, 0.021)	0.020 (0.019, 0.021)	0.51
Female	0.05 (0.04, 0.06)	0.06 (0.05, 0.07)	0.05 (0.04, 0.06)	0.22
Female - Undetected	0.018 (0.016, 0.019)	0.017 (0.016, 0.019)	0.018 (0.017, 0.019)	0.88
Race/ethnicity:				
Non-Hispanic White	0.05 (0.04, 0.06)	0.07 (0.05, 0.08)	0.05 (0.04, 0.06)	0.14
Non-Hispanic White - Undetected	0.018 (0.016, 0.020)	0.018 (0.017, 0.019)	0.19 (0.018, 0.020)	0.66
Non-Hispanic Black	0.16 (0.13, 0.19)	0.14 (0.10, 0.19)	0.11 (0.09, 0.15)	0.11
Non-Hispanic Black - Undetected	0.23 (0.020, 0.030)	0.22 (0.020, 0.024)	0.22 (0.021, 0.024)	0.88
Mexican American	0.06 (0.04, 0.08)	0.05 (0.04, 0.07)	0.05 (0.04, 0.06)	0.40
Mexican American - Undetected	0.18 (0.017, 0.020)	0.18 (0.016, 0.020)	0.17 0.016, 0.019)	0.55

**Figure 2:**
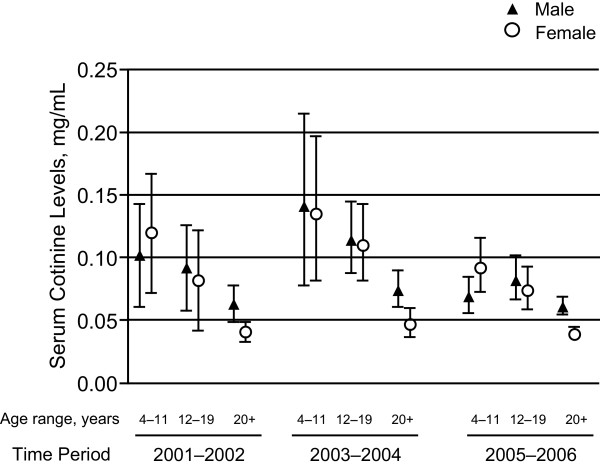
**Geometric means and 95% confidence intervals for nonsmokers in the United States by age and gender, 2001-2002, 2003-2004, and 2005-2006**.

**Figure 3:**
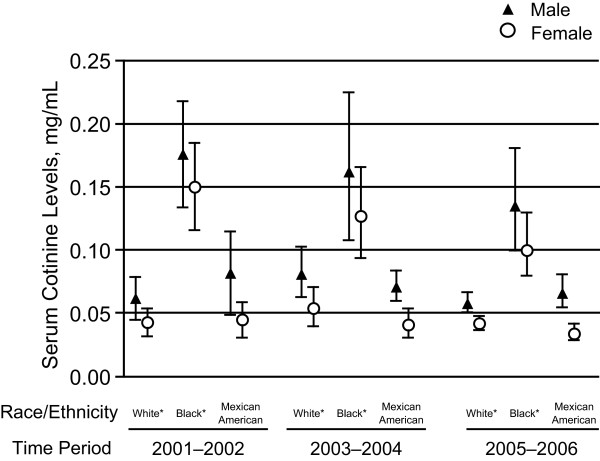
**Geometric means and 95% confidence intervals for nonsmokers in the United States by race/ethnicity and gender, 2001-2002, 2003-2004, and 2005-2006**. *Non-Hispanics.

**Figure 4:**
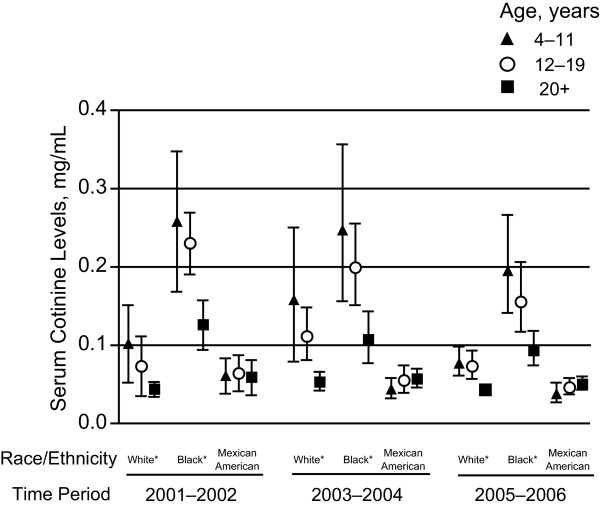
**Geometric means and 95% confidence intervals for nonsmokers in the United States by race/ethnicity, 2001-2002, 2003-2004, and 2005-2006**. *Non-Hispanics.

The adjusted geometric means and 95% CIs for 2-way interaction over the 3 time periods are presented in Figure 2, Figure 3, Figure 4. The results of a 2-way interaction between age and gender are presented in Figure 2. The noted age effect, children appearing to have higher cotinine levels than adults, is observed in both males and females. During 2003-2004, cotinine levels showed a notable increase across all 3 age groups since 2001-2002, but these increases seem to have disappeared by 2005-2006. The noted gender effect, males appearing to have higher cotinine levels than females, is observed across all age groups over time, except in children aged 4-11 years old during 2001-2002 and 2005-2006.

The results of a 2-way interaction between race/ethnicity and gender are presented in Figure 3. The noted race/ethnicity effect, non-Hispanic Blacks showing higher cotinine levels than non-Hispanic Whites and Mexican Americans, was consistent across gender groups over time. Males also consistently showed higher cotinine levels than females across the race/ethnicity groups. During all 3 time periods examined, both male and female non-Hispanic Blacks displayed notably higher cotinine levels than their respective comparison groups.

The results of a 2-way interaction between age and race/ethnicity are presented in Figure 4. The noted age effect of children at higher risk of SHS exposure is distinctly visible among non-Hispanic Whites and non-Hispanic Blacks. This age effect is not visible among Mexican Americans across all 3 time periods and becomes less visible among non-Hispanic Whites by 2005-2006.

## Discussion

This study reports updated findings using the latest available NHANES data between 2001 and 2006. Overall, our results suggest that the long-term trend of declining exposure to SHS among nonsmokers, as reported by Pirkle et al. [6], have stabilized since 2002. While the work of Pirkle et al. provided evidence to show the progress and impact of antismoking policies and public health efforts over the last decade, our current findings reveal that declines in SHS exposure seem to have leveled off.

Despite the stabilizing downward trend in national exposure, the demographic and racial/ethnic differences noted by Pirkle et al. [6] persist today, with the young (children and adolescents age 4-19 years), males, and non-Hispanic Blacks experiencing higher levels of SHS exposure. While male nonsmokers were consistently exposed to higher cotinine levels than female nonsmokers, their overall cotinine levels remained consistently below 0.1 ng/mL over time. Children and adolescents age 4-19 years of non-Hispanic Black descent had consistently higher cotinine levels than their comparison groups across the 3 time periods examined. During 2005 to 2006, overall cotinine levels in children age 4-11 years fell below 0.1 ng/mL. While there is no direct evidence from this study to address the possibility of a declining trend in children's exposure, this finding is consistent with the Healthy People 2010 objective calling for no more than 45% of nonsmokers to have cotinine levels >0.1 ng/mL [6].

In children, our findings are consistent with previous Centers for Disease Control and Prevention publications concluding that children are more heavily exposed to SHS than nonsmoking adults. Specifically, children's measured cotinine levels have been twice that of adults [9]. The reasons for this are multifactorial. Children have a ventilation rate that is higher, proportional to their body size, than that seen in adults. The toxicity level in SHS is in effect amplified in children as they inhale many of the same cancer-causing substances as current smokers [10]. In addition, children get most of their smoke exposure in private homes and vehicles [11]. Children can not choose who they live with, whereas nonsmoking adults can choose to live with nonsmokers or have people smoke outside the home. There is currently little data to inform on the specific impact of controlling private household or vehicle exposure.

Concerning racial and ethnic differences in exposure, our findings are consistent with previous studies concluding that Black nonsmokers have higher serum cotinine levels than do White or Mexican American nonsmokers [6]. It is also consistent with previous studies that documented differences between Black and White smokers [12,13].

One question that these data raise is whether ethnic/demographic differences in African Americans, males, and children are due to their differences in nicotine metabolism. In 1998, Pérez-Stable et al. [12] reported that African Americans take in more nicotine per cigarette than whites, which may explain why African Americans have a higher incidence of lung cancer. In general, African Americans have a slower rate of nicotine metabolism than Caucasians, resulting in greater accumulation of cotinine.

The results from the 2-way interaction between age and gender are notable, showing the highest exposure levels in children and adolescents age 4-19 years of non-Hispanic Black descent as compared with other comparison groups over time. This finding is consistent with previous studies linking a lower socioeconomic status to a greater probability of SHS exposure in children. Previous research has suggested that persons with low income are more likely to be exposed in the home than are other income groups. Specifically, 58% of children in households with annual incomes under $10,000 per year experience SHS exposure, as compared with 30% of children in households with annual incomes greater than $40,000 [14]. It has been previously documented that African Americans earn less money per year and have the highest poverty rates as compared with other race/ethnicity groups [15].

Our study has some strengths and limitations that should be noted. The data used in this analysis comes from a large national sample of individuals who are representative of the United States civilian non-institutionalized population. To validate the differences and/or similarities that are observed over time, consistent methodology was utilized across all time periods examined. One important caveat to consider in understanding the current study findings is the tool being used to characterize exposures over time. While cotinine represents the most widely used biomarker (primarily due to its specificity, half-life, and ease of measurement), questionnaire reports remain the primary tool to track exposures over a certain time period. Previous findings from questionnaire reports have been compared with biomarker data and have demonstrated high degrees of correlation between the two ways of exposure assessment [16]. In addition, this study is currently missing an in-depth analysis of chronic conditions (e.g., asthma attacks and lung cancer) given infrequent observations within a single wave of data. Such information would better inform and substantiate our understanding of the relationship between serum cotinine levels and impact on various health risks.

Important GAPS and research questions remain concerning the progress and impact of reducing SHS exposure. Despite the relatively widespread implementation of smoke-free laws in the US, there are opportunities for states to implement greater protection. We now know that only 100% smoke-free indoor air laws can effectively protect the public from secondhand smoke. Yet, as of the end of 2008, only 16 states have provided 100% smoke-free indoor air laws for bars, restaurants, government worksites, and private worksites [17]. The extent of smoke-free legislation was likely even less comprehensive two years prior, during which the data for the current analysis was captured (2001 - 2006). It is possible that an analysis using post-2006 data may demonstrate improvements though these data were not available at the time of the current study. In addition, indoor spaces such as homes and vehicles represent another critical area of focus with regard to reducing a major location of exposure.

Finally, it is possible that the measurement of secondhand smoke exposure using serum cotinine analysis is approaching its limits of detection and possibly introducing challenges to demonstrate further declines in exposure. Further efforts of continuous method improvements could lead to more sensitive instruments that will serve to be even more effective in monitoring and detecting further exposure declines.

## Conclusions

Our findings reflect long-term declining trends in SHS exposure among nonsmokers to be stabilizing. These findings could reflect opportunities for implementing greater protection. Since there are no risk-free levels of SHS [1], previous studies have established that the most effective approach to protect nonsmokers is to implement smoke-free policies that completely eliminate exposure. While some states and locales are implementing smoke-free policies and legislation, the parameters are generally not uniform and vary widely by geographical region, occupation, and industry[18]. It would also be important to target efforts on subgroups that are persistently at-risk for high SHS exposure, namely the young, males, and non-Hispanic Blacks. Finally, it will be necessary to introduce more sensitive instruments capable of detecting incremental declines in SHS exposure.

## Abbreviations

CI: confidence intervals; NHANES: National Health and Nutrition Examination Surveys; SHS: secondhand smoke.

## Competing interests

CC, CB, and VM are employees of Pfizer, Inc.; TB is an employee of Abt Bio-pharma Solutions, Inc., who were paid by Pfizer, Inc., to carry out the statistical analysis in connection with the development of this manuscript; DM was a paid consultant to Pfizer, Inc. in connection with the development of this manuscript.

## Authors' contributions

All co-authors contributed to the conceptualization of the paper. TB conducted the statistical analyses. All co-authors assisted with the analyses and revised drafts.

## Pre-publication history

The pre-publication history for this paper can be accessed here:

http://www.biomedcentral.com/1471-2458/10/359/prepub
